# Navigating ethical, regulatory, and implementation barriers to AI in healthcare: pathways toward inclusive digital health in low-resource settings—a scoping review

**DOI:** 10.3389/fdgth.2026.1763884

**Published:** 2026-04-13

**Authors:** Areeba Shahid, F. N. U. Nisha, Zaira Azhar, Tehreem Rauf, Elaf Eman Ishaq, Gaurav Kansal, Malik Olatunde Oduoye, Oluwapelumi Wumi Adeniji, Joseph Conteh

**Affiliations:** 1 Department of Medicine and Surgery, Jinnah Sindh Medical University, Karachi, Pakistan; 2 Department of Medicine and Surgery, Liaquat University of Medical and Health Sciences, Jamshoro, Pakistan; 3 Department of ML &C, Govt of Pakistan, Ministry of Defence, Rawalpindi, Pakistan; 4 Department of Medicine and Surgery, Fatima Jinnah Medical University, Lahore, Pakistan; 5 Department of Medicine, Watim Medical College, Islamabad, Pakistan; 6 Department of Medicine and Surgery, Government Medical College, Patiala, India; 7 Department of Research, The Medical Research Circle (MedReC), Goma, Democratic Republic of Congo; 8 Department of Applied Health, Brigham Young University, BYU Pathway Worldwide, Rexburg, ID, United States; 9 School of Public Administration, The University of New Mexico (UNM), Albuquerque, NM, United States

**Keywords:** artificial intelligence, developing regions, digital health, ethics, health equity, regulation

## Abstract

**Background:**

Artificial intelligence (AI) has the potential to revolutionize healthcare delivery in low- and middle-income countries (LMICs), yet its rapid adoption raises complex ethical, regulatory, and implementation challenges. This review investigates these barriers and identifies emerging strategies that support equitable and inclusive AI deployment in resource-limited settings.

**Methods:**

Following the PRISMA Extension for Scoping Reviews (PRISMA-ScR) guidelines, a systematic mapping of literature was conducted using PubMed, Scopus, and Cochrane Library (2000–2025) alongside global health policy reports. The search was framed using the Population, Concept, and Context (PCC) framework to identify studies addressing AI governance in LMICs. A total of 60 sources addressing ethical, regulatory, or implementation issues were analyzed across three domains derived from the WHO and OECD frameworks: governance, privacy, and AI applications.

**Results:**

This study reveals that 7.4% of LMICs have adopted national AI strategies. Evidence indicates that over 60% of AI models in LMICs rely on non-representative datasets, increasing contextual bias. Of the 60 included studies, 25 focused on ethics, 17 on regulatory gaps, and 18 on implementation. Findings highlight workforce readiness gaps, with fewer than 10% of institutions offering structured AI training. Case studies from Brazil and India illustrate how these barriers are addressed through context-sensitive design.

**Conclusion:**

Successful AI integration requires context-sensitive design, participatory governance, and capacity building. This scoping review identifies critical gaps in empirical research on operationalization and recommends a transition from digital dependency to local innovation ecosystems.

## Highlights

- A significant majority of LMICs lack formal national AI strategies, creating a critical gap in regulatory oversight and ethical governance.
- Over 60% of AI tools deployed in LMICs rely on high-income country datasets, while locally trained models show 25%–30% higher predictive validity.
- Fewer than 10% of reviewed LMIC health institutions provide structured AI training, highlighting workforce and infrastructure deficits that hinder sustainable integration.

## 1 Introduction

Artificial Intelligence (AI) refers to a set of technologies that allow machines and computers to simulate human intelligence (1). AI has emerged as a transformative force in various sectors, with healthcare being one of the most significant. The integration of AI into hospitals and clinics represents a paradigm shift in how medical care is delivered and managed (2). AI could transform physician workflow and patient care through its applications, from assisting physicians and replacing administrative tasks to augmenting medical knowledge (3).

Developing countries refer to countries in which the Gross National Income (GNI) per capita per year does not exceed $11,905. In developing countries, the life expectancies and health status of rural residents are generally worse than those of urban residents. Poverty is one of the biggest social determinants. Limited access to qualified healthcare is the immediate cause of poor health status. Low public health spending, low coverage of health insurance, a limited benefit package, shortages of health professionals and facilities, lack of training for health workers, transportation difficulties, and so on all contribute to the low quality of healthcare in rural areas of developing countries. In developing countries, the inequality between urban and rural health services is a serious problem, of which the shortage of qualified healthcare providers is the major cause of the unavailability and low quality of healthcare in rural areas.

Research has shown that the application of computer-assisted or AI medical techniques could improve healthcare outcomes in rural areas of developing countries (4). Concurrent advances in information technology infrastructure and mobile computing power in many low and middle-income countries (LMICs) have raised hopes that AI might help to address challenges unique to the field of global health and accelerate achievement of the health-related sustainable development goals. A series of fundamental questions has been raised about AI-driven health interventions, and whether the tools, methods, and protections traditionally used to make ethical and evidence-based decisions about new technologies can be applied to AI (5). Indeed, while AI has the potential for great benefit through its main purpose of helping people lead longer and flourishing lives, it has the capacity to be misused or abused for evil and harm. Examples of this include covert surveillance systems used by authoritarian regimes to oppress dissents, militarized killer robots, and discrimination and bias in the data fed to the programs in training, as this is translated to discrimination in AI decision-making.

Other concerns include AI leading to a lack of privacy, transparency, misuse of personal data, and potential loss of informed consent as data can be collected secretively; a loss of human contact and empathy, and a lack of accountability and liability (6). Given the interdisciplinary, normative, and policy-driven nature of these challenges, a scoping review approach was chosen to synthesize ethical, regulatory, and implementation perspectives across diverse contexts.

## 2 Methodology

### 2.1 Study design and rationale

This study employed a scoping review design to examine data governance and privacy in AI-enabled healthcare systems in LMICs. A scoping review was selected because it is the most appropriate method for mapping the nature and extent of evidence in an emerging, multidisciplinary field. This study was conducted in accordance with the Preferred Reporting Items for Systematic Reviews and Meta-Analyses extension for Scoping Reviews (PRISMA-ScR) guidelines (7). Unlike a narrative review, this approach follows a systematic protocol to identify research gaps and organize heterogeneous evidence—including policy documents and peer-reviewed studies—that cannot be pooled quantitatively.

### 2.2 Search strategy and eligibility (PCC framework)

To ensure methodological rigor, the study was guided by the PCC (Population, Concept, and Context) framework:

- Population: Healthcare systems, providers, and patients in resource-limited settings.
- Concept: Ethical, regulatory, and implementation barriers to inclusive AI.
- Context: Low- and Middle-Income Countries (LMICs).

Searches were performed in PubMed, Scopus, and the Cochrane Library (2000–2025). The 25-year range was purposefully selected to provide a longitudinal baseline, capturing the transition from early e-Health infrastructure to the recent surge in Generative AI. While the search window is broad, the final inclusion of 60 studies reflects the extreme scarcity of formal governance literature in LMICs before 2015. Most historical records from 2000 to 2010 focused on digitizing health records rather than the ethical governance of autonomous algorithms, which explains why the majority of the results were excluded during full-text review for lacking a policy or governance focus. Grey literature from the WHO, World Bank, and OECD was included to capture global standards.

Conceptual Framework Findings were organized under three domains—governance, privacy, and AI applications—derived from the WHO Ethics and Governance of AI for Health and OECD AI Principles. This framework ensures consistency with global standards and provides a structured lens for synthesizing diverse evidence types.

### 2.3 Study selection and data synthesis

A total of 60 studies were included in the final synthesis. This final count was rigorously verified to ensure consistency across the results and discussion. Data extraction focused on identifying context-specific barriers and “missing” outcomes in current LMIC policy. In accordance with scoping review standards, a formal quality appraisal of individual studies was not conducted, as the goal was to map the breadth of the field rather than verify specific clinical effects.

## 3 Results

### 3.1 Selection of sources of evidence

**Figure 1:**
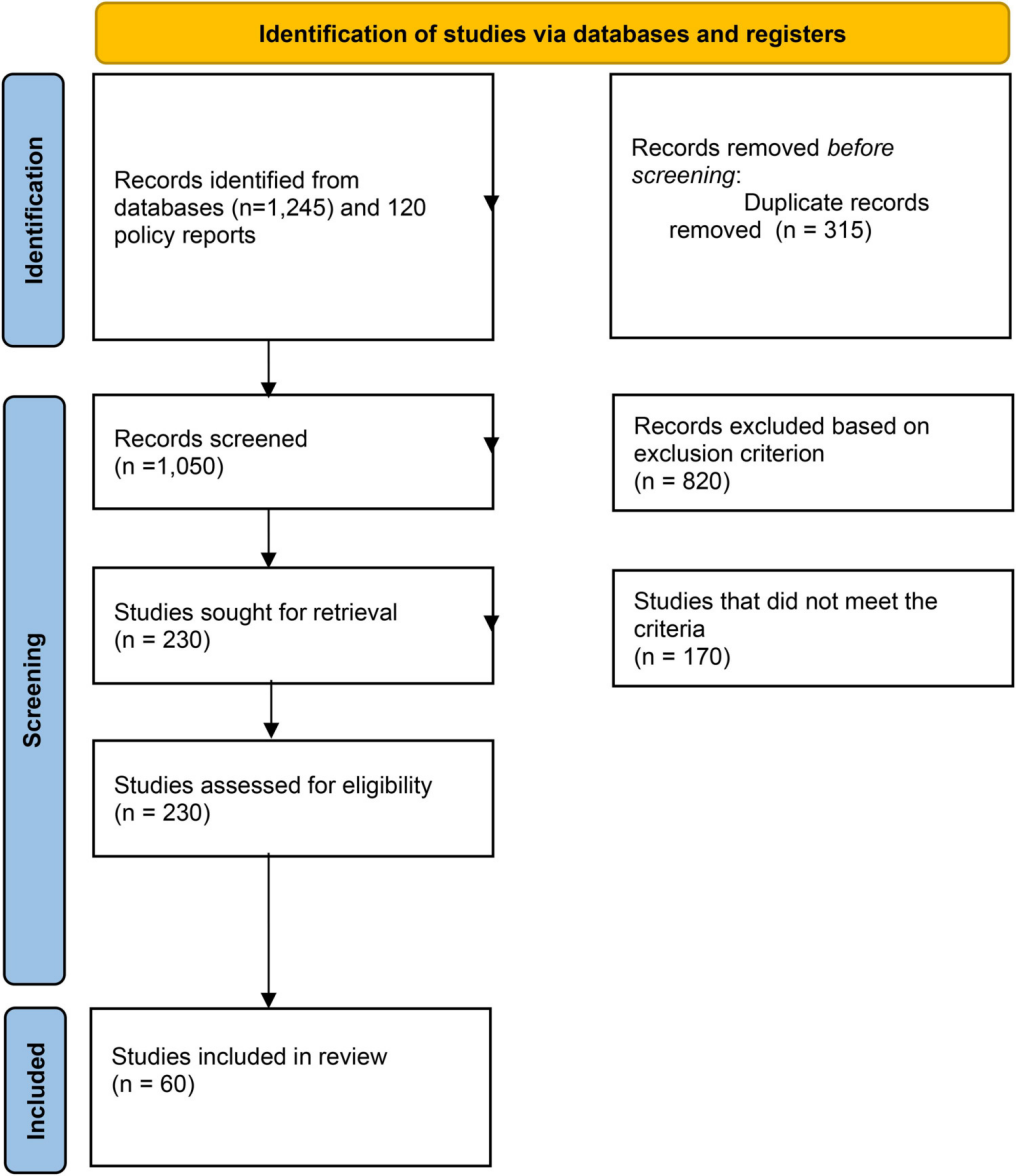
PRISMA chart.

The initial search across four databases and grey literature sources yielded a total of 1,365 records (1,245 from databases and 120 from policy reports). After the removal of 315 duplicate records, 1,050 unique citations remained for title and abstract screening. During the initial screening phase, 820 records were excluded based on pre-defined exclusion criteria, such as a lack of focus on healthcare or non-LMIC contexts. This resulted in 230 sources sought for full-text retrieval and eligibility assessment. Upon full-text review, 170 studies were excluded because they did not meet the specific inclusion criteria—primarily due to a focus on purely technical algorithmic descriptions without governance analysis or being published before the year 2000. Ultimately, 60 studies met all criteria and were included in the final scoping review synthesis. The systematic selection process is illustrated in the PRISMA flow diagram (Figure 1). Detailed characteristics and the thematic focus of each of the 60 included sources are provided in Supplementary Table 1.

### 3.2 Characteristics of included sources

Of the 60 included sources (summarized in Supplementary Table 1), the majority were published between 2018 and 2024, reflecting the recent surge in AI applications in global health. Geographically, the evidence was distributed across Sub-Saharan Africa, South Asia, and Latin America. In terms of design, the review included a mix of peer-reviewed empirical research, policy frameworks, and case reports.

## 4 Synthesis of results

Findings from the 60 sources were organized under three domains: governance, privacy, and AI applications, derived from the WHO and OECD digital health governance frameworks. Within this corpus, 25 studies addressed ethics and governance, 17 addressed regulatory frameworks, and 18 focused on implementation and AI applications. This structured synthesis ensures alignment with global standards for digital health oversight.

### 4.1 Ethical challenges in AI-driven health systems

#### 4.1.1 Algorithmic bias and health inequities

Among the key limitations in implementing AI in healthcare is the underrepresentation of various populations in training datasets, thereby exacerbating existing social and ethnic disparities. This compromises model accuracy and reliability across diverse patient groups. Evidence from global reviews shows that assurance of data diversity and equity-focused design can reduce systemic biases in healthcare delivery (8, 9).

#### 4.1.2 Data governance and privacy

Obtaining informed consent is often difficult in low-literacy settings due to linguistic, educational, and technological barriers (10). Additionally, AI use in cross-border health data transfers presents a significant risk because of privacy laws and weak safeguards. Without harmonized regulations or enforcement capacity, populations in low- and middle-income countries face an increased risk of misuse or exploitation (11).

#### 4.1.3 Transparency and accountability

Although AI's role in healthcare has shown great potential to enhance clinical decision-making, accountability remains a major challenge. The multiple roles of developers, clinicians, and patients make it difficult to assign responsibility. Current legal and regulatory frameworks lack a clear definition of accountability, especially when decisions are made by opaque or black box algorithms. As a result, this lack of clarity impairs transparency and ethical use of AI in healthcare (12). Additionally, opaque AI models increase legal and moral uncertainty, making it hard to assign accountability for adverse outcomes (13). Table 1. Ethical Challenges in AI-Driven Healthcare Systems in LMICs.

**Table 1:** Ethical challenges in AI-driven healthcare systems in LMICs.

Ethical Domain	Key Issues Identified	Implications for LMICs	References
Algorithmic bias	Underrepresentation of local populations in training datasets	Exacerbates existing health inequities; limits model accuracy	Celi et al. (8); Nazer et al. (9)
Data governance & privacy	Weak data protection and difficulties obtaining informed consent	Increased risk of data misuse and exploitation	Fletcher et al. (10); Hewarathna (11)
Transparency & accountability	Opaque “black box” models and unclear liability	Reduces trust; creates legal uncertainty	Kiseleva et al. (12); Smith (24)

### 4.2 Regulatory and policy challenges

#### 4.2.1 Absence or weakness of national AI strategies

Among the many central challenges in the adoption of AI in developing countries is the lack of a comprehensive national strategy for AI in health. While many high-income countries have developed clear national AI frameworks, many middle- and low-income countries lack guiding frameworks and strategic planning to guide responsible deployment (14, 15). According to the policy analysis report as of 2022, only 7.4% of LMICs have adopted formal national AI strategies, with adoption particularly low in Africa (only 4 of 54 countries) (16, 17), while only 15.2% of the global countries have AI-specific legislation, with around 60.3% of the global nations lacking an infrastructure of AI in healthcare (18). While several LMICs are still developing AI-specific laws, existing frameworks such as South Africa's Protection of Personal Information Act (POPIA) (19) and India's Digital Personal Data Protection Act (2023) (20) currently provide the primary legal basis for the Privacy Domain in these regions. Moreover, the adoption of AI models trained on data from high-income countries often fails to capture the unique context of low- and middle-income countries, leading to poor validity, biases, and inequitable outcomes. The absence of infrastructure and validation frameworks increases the risk (21, 22).

#### 4.2.2 Incoherence between health and tech policy

Governance is the essential framework for ensuring that AI technologies are ethical, transparent, accountable, and adaptable. Although many states have implemented system-wide AI governance policies, there remains a lack of a robust governance system (23). Many studies recommend that governance should be multidisciplinary, involving clinicians, ethicists, legal advisors, and patients. These policies include ongoing auditing and risk assessment (24, 25).

#### 4.2.3 Liability and accountability gaps

The lack of transparency and autonomous decision-making creates a responsibility gap, making it unclear who should be held accountable for patient harm, whether the clinician, the developer, the institution, or the regulator. This uncertainty undermines reliability and legal certainty, particularly when it comes to the adoption of such opaque systems in low-resource contexts (26, 27), underscoring the need for robust regulatory frameworks that supersede liability and establish clear care compliance to create transparency (28). Table 2. Regulatory and Policy Barriers to AI Implementation.

**Table 2:** Regulatory and policy barriers to AI implementation.

Barrier Type	Findings	Examples/Data	References
Lack of national AI strategy	Absence of comprehensive governance structures	Only 4 of 54 African nations have national AI health strategies	Leam (16)
Fragmented governance	Poor coordination between the health and technology sectors	Limited cross-ministerial planning	Ferlito et al. (23)
Liability gaps	Undefined accountability for harm	No clear delineation of clinician vs. developer responsibility	Maliha et al. (25); Kim et al. (28)

### 4.3 Implementation challenges

Despite showing great potential in global health, the implementation of AI and digital health encounters various technical, scientific, infrastructural, ethical, and sociocultural challenges, especially in LMICs. Table 3. Implementation Challenges in LMICs.

**Table 3:** Implementation challenges in LMICs.

Challenge Area	Description/Impact	Examples	References
Infrastructure deficits	Weak internet, electricity, and computing capacity	An AI diabetic retinopathy tool in Zambia is limited by connectivity	Bellemo et al. (29)
Workforce capacity	Lack of AI training and technical support	Jordanian hospitals cited poor training as the main barrier	Albahar et al. (32)
Sociocultural factors	Distrust, language, and cultural mismatches	Job displacement fears in Africa; privacy concerns in Latin America	Frimpong (37)

#### 4.3.1 Infrastructure deficits

One of the major barriers in LMICs is resource limitation. AI technologies are not prioritized for resource allocation due to scarce resources and financial constraints in LMICs (21), directly impacting their development, design, and integration into healthcare. While evaluating the accuracy of an AI model in screening for diabetic retinopathy in Zambia, Bellamo identified two main barriers in its implementation: affordability and usability. For an under-resourced setting, even beneficial technologies like AI may become impractical to use due to financial cost, the absence of an optimal telecommunication network (29), lack of internet availability, and shortage of electricity (30).

This is further compounded by the financial cost of continuously upgrading software algorithms and testing, as well as monitoring AI systems to ensure the functioning of all hardware components. With the recent advancements in AI healthcare, initial investments from governments, industries, and academia are increasing; however, the long-term sustainability of AI systems remains uncertain. Sufficient business incentives are required for the adoption and optimization of this technology; however, without sustainable financial models, even the most promising technologies can fail to deliver an impact (31). The successful deployment of an AI-driven technology requires extensive investment to strengthen resources, infrastructure, and potential training to support new interventions.

#### 4.3.2 Workforce and capacity gaps

Insufficient AI training for health professionals is another key challenge in AI-driven healthcare in LMICs. This gap in knowledge and education extends not only to clinicians but also to policy-makers, AI developers, and even individual patients. Lack of collaboration among them presents a significant barrier to the effective adoption and understanding of these technologies. A study by AlBahar et al. identified lack of appropriate training followed by lack of access to technical support as the major challenges in the implementation of clinical decision support systems (CDSS) in Jordanian Hospitals (32). Insufficient training results in the creation of a workforce that is unable to manage or effectively use AI tools, which emphasizes the importance of adequate training and expertise in understanding these technologies (33).

Time-intensive tasks like collecting and labelling data might also pose barriers, especially considering the busy schedules of the healthcare staff (34). Effective implementation of AI in medicine also requires clinicians' trust and acceptance of AI into the existing healthcare systems. AI technology may face resistance due to the fear that it will replace healthcare professionals in the future (21). Many healthcare professionals may also find it difficult to trust automated systems due to insufficient training. This lack of familiarity and understanding may lead to difficulty in relying on AI solutions. 22 clinicians in rural China, interviewed by Wang et al., raised their doubts about AI-powered CDSS, such as misalignment with the local context and transparency and trustworthiness of this system (35). Another study in China indicated that some of the users considered the diagnosis produced by AI-Chatbots as inaccurate (36).

#### 4.3.3 Sociocultural and linguistic barriers

Frimpong presented a comprehensive overview of the cultural influence on the use of AI across various countries. In Africa, major concerns related to AI included job displacement and socioeconomic divide. In South Africa and Kenya, AI development raised concerns like increased dependency on Western powers, privacy violations, and the risk of exposure of local data to foreign countries (37). In Latin America, there's widespread apprehension about government surveillance, privacy, and human rights violations. In Brazil, political corruption and distrust in government further impact the cultural acceptance of AI, alongside the fears that AI could exacerbate gender inequalities, marginalize communities, and negatively impact indigenous languages (37).

A significant challenge for AI implementation is language limitations. A single medical word might have different meanings in different contexts. Even if an AI system identifies different meanings of a single word, it may still struggle to determine the context in which it occurs (38). To solve this problem, the AI must be trained on the data, including a diverse local population, and must be adopted into the local language to better understand the context.

Using colonoscopy as an example, Carrell et al. described various challenges associated with applying natural language processing (NLP) systems to heterogeneous settings. Major challenges highlighted included difficulties in building natural language corpora, different record structures, and idiosyncratic linguistic content (39). This would likely be multiplied in low-resource settings, where hand-written health records further complicate the assembling of usable natural language corpora (40). Furthermore, recent developments in Large Language Models (LLMs) have introduced additional risks regarding “hallucinations” and a lack of local linguistic representation in LMICs (41), necessitating robust validation of generative AI tools within the specific sociocultural contexts of low-resource systems.

## 5 Discussion

This scoping review synthesizes ethical, regulatory, and implementation insights across diverse LMIC contexts, emphasizing conceptual coherence rather than exhaustive evidence coverage. The study found that AI has remarkable potential to show transformation across many domains of healthcare, particularly in LMICs, where healthcare systems are underserved and overloaded. AI technologies are increasingly being leveraged in resource-constrained settings to bridge critical gaps in healthcare service delivery, diagnosis, and disease surveillance. AI and telemedicine integration present a promising solution to healthcare access in rural areas by providing access to advanced diagnostic tools, predictive analytics, and personalized treatment recommendations, thus empowering local providers with decision support and hence reducing delays and improving treatment outcomes, also reducing unnecessary clinical visits and easing facility burden (42).

AI-driven medical diagnostics have replaced traditional systems due to their cost-effectiveness and minimal reliance on infrastructure, equipment, and specialized personnel. Furthermore, a faster diagnostic process, along with a high degree of accuracy, can be achieved (43). In terms of disease surveillance, AI enables early detection, real-time monitoring, and efficient resource allocation by analyzing data from multiple sources, such as search trends and electronic health records, to predict early disease outbreaks and issue automated alerts (44).

The use of AI predictive analytics has been increasingly applied in the control of infectious diseases. The integration of machine learning models with epidemiological and meteorological data to predict dengue outbreaks has allowed public health authorities to take necessary and preemptive actions and to allocate resources more effectively, particularly in vulnerable areas (45).

Among the crucial considerations in the application of AI in healthcare settings in low and middle-income countries is the clear distinction between locally developed and imported AI tools. Locally tailored tools are built on region-specific data, align with local healthcare infrastructure and disease profiles, providing superior explainability, contextual fit, and cost-efficiency, particularly among low-resource settings. In contrast, imported tools adapted from high-income countries are based on data sets and clinical workflows that do not translate well to low and middle-income countries, hence raising concerns about data compatibility and model generalizability (46, 47).

### 5.1 Linking results to inclusive principles

The thematic clusters identified in the Results, Ethical (3.1), Regulatory (3.2), and Implementation (3.3), show a cycle of digital dependency in LMICs. To move from these barriers toward a functional framework, we propose four **Principles of Inclusive AI**. These principles are not independent of the results; rather, they are direct responses to the data. For instance, the “Context Sensitivity” principle (4.2.1) is the necessary response to the “Algorithmic Bias” (3.1.1) and “Contextual Bias” (3.2.1) found across the reviewed literature. Similarly, “Open Source and Frugal Innovation” (4.2.3) provides a strategic pathway to overcome the “Infrastructure Deficits” (3.3.1) identified in studies like Bellamo's work in Zambia (29).

### 5.2 Principles of inclusive and equitable AI

#### 5.2.1 Context sensitivity

Context sensitivity in AI refers to the adaptation and effective integration of AI models to local sociocultural, disease patterns, and existing health systems. This ensures that the AI solutions are appropriate, i.e, the AI solutions and local population are matched in terms of demographics and disease characteristics (10). A significant issue arises when AI models trained with HICs data are deployed in LMICs, thus impacting their adoption and effectiveness in clinical practice. This phenomenon is also referred to as contextual bias, which is defined as the deployment of algorithms created in HICs in LMICs with different health contexts, population demographics, health risks, and treatment decisions (48). A study evaluated the effectiveness of an HIC-made AI model in a hospital in Vietnam and found that the model's performance significantly declined as it was employed in a different setting from where it was trained (46). Therefore, it is necessary to build AI models trained on algorithms using local datasets. It ensures relevance, effectiveness, and usability in diverse settings.

To ensure context sensitivity, collaboration among academia, clinical practice, and industry is necessary to create models that are both technically sound and clinically relevant. Local stakeholders must be involved in data collection, validation, and regulation, as well as in the development of the legal framework (21).

#### 5.2.2 Participatory design

All relevant stakeholders, including healthcare professionals, AI experts, and community members, must be involved in the design and implementation of AI solutions. A synergistic effort is required by both physicians and AI developers to optimize the AI tools in clinical settings. Physicians offer a better understanding of diseases, symptoms, and patients' healthcare needs, while AI developers possess the technical skills required for designing and implementing an AI tool. A multidisciplinary approach will result in a tool that is more effective and viable in the clinical world (49).

With the incorporation of AI into medicine, it has become necessary for physicians to develop essential skills like data analysis, statistics, and AI ethics. To educate health professionals in AI, medical school and residency curricula must be updated to include training in comprehension of algorithms and datasets, and informatics (31). Training local AI talent is necessary to ensure that the technology meets the demands and needs of local social and cultural norms. Strategic partnerships must be established between HICs and LMICs, with HICs providing funding and investment to ensure the implementation of AI in LMICs (50).

#### 5.2.3 Open source and frugal innovation

Considering the infrastructure gaps in LMICs, possible areas of focus should include investment in strong internet networks, provision of electronic health records, and construction of national eHealth infrastructure. To combat increasing demand for computing power and capacity, join programs and access funding from IT companies. An agreement should be signed with local and international software developers to offer open licensing and free training of their products to reduce the financial cost and educational barrier. Strategic partnerships are required between HICs and LMICs around open-source platforms, mobile applications, and digital health for more cost-effective solutions. National research and innovation should be encouraged to generate AI solutions that are culturally appropriate and socially acceptable (21).

EMRs, the digital versions of patient and population health information, can help solve the issue of data acquisition and storage in LMICs. OpenMRS is one such system being used in Kenya to improve the completeness of maternal and child health data (51). Cloud computing is another cost-effective application for data storage and processing. By using remote servers, LMICs are able to access previously unattainable computing power. It facilitates the AI implementation even in settings that lack adequate IT infrastructure. Widespread use of mobile phones in LMICs also helps achieve health objectives and deliver targeted health information in an under-resourced setting (40).

The OpenMRS implementation reflects the Governance domain and Principle 4.2.3 (Open Source Innovation) by building a sustainable national eHealth infrastructure.

#### 5.2.4 Local innovation ecosystems

Local innovative ecosystems include the local AI talent, industries, research setups, clinicians, government, and policy makers. A strong local ecosystem enables AI development aligned with national interests and local health priorities. It reduces dependency on external powers, creating employment opportunities, building local capacity, and implementing context-sensitive interventions. Alami et al. also emphasized the involvement of responsible local leadership, inclusive of all stakeholders, as one of the five building blocks of sustainable and inclusive AI, stating that fostering effective collaborations among them is necessary to develop robust AI technology (14).

Contract agreements with HICs can also help facilitate education and training, empowering local clinicians' capabilities through the development of new professional societies (21).

### 5.3 Role of civil society and patients

Civil society, individual patients, and community groups play an active and invaluable role in all stages of AI development and deployment. Bazzano described how AI can be a powerful tool in public health, given the participatory methods and community engagement. Community engagement is vital for equitable distribution of AI and preventing harm and promoting transparency. This engagement ensures that AI doesn't precipitate health disparities and avoids the risks of bias (52).

Public input in AI is essential for promoting transparency and effectively mitigating the harmful impact of AI; however, insufficient understanding of how it works, its ability to evolve, and resource requirements might hamper the meaningful involvement of society and individuals. Sieber et al. categorized the public participation in AI into five distinct themes: Participation as a natural byproduct of automating government, facilitated through an AI medium, as a process of quantification, as a technocracy of trust, and as truly meaningful engagement. These five themes describe various approaches to civic engagement in AI. Participation may naturally arise as a byproduct of government automation or as AI tools and chatbots gather public data, facilitating participatory design. It may often be reduced to quantifiable data or, more importantly, build trust and gain acceptance for AI by specific references to transparency, accountability, open data literacy, and privacy. The last theme calls for a more genuine and aspirational approach, i.e, focusing on empowering citizens, co-design, and co-production (53). This grassroots engagement is essential to mitigate the transparency and accountability gaps (Section 4.1.3) identified in our review of current LMIC frameworks. (See Table 4. Inclusive AI Development: Guiding Principles and Actions).

**Table 4:** Inclusive AI development: guiding principles and actions.

Principle	Description	Actionable Strategies	Expected Outcome
Context sensitivity	Adapt AI to local disease patterns and sociocultural contexts	Use local datasets and region-specific validation	Improved accuracy and trust
Participatory design	Engage clinicians, communities, and developers collaboratively	Multidisciplinary design and training programs	Enhanced usability and ethical adoption
Open-source innovation	Promote affordable and adaptable systems	Support open licensing and frugal innovation	Reduced cost barriers
Local innovation ecosystems	Build national capacity and AI talent	Encourage partnerships and regional centers of excellence	Sustainable and contextually relevant AI systems

This approach is a critical response to the “black box” opacity (Section 4.1.3) and responsibility gaps identified in our results. By moving toward meaningful engagement, LMICs can transition from being passive recipients of imported technology to active participants in an equitable local innovation ecosystem.

## 6 Case examples of principles in action

To validate the framework proposed in the discussion section, the following cases illustrate how theoretical principles are operationalized to solve real-world implementation barriers.

### 6.1 Brazil: predictive outbreak management

Brazil is home to about half of all reported dengue cases in the Americas. In the 3 decades after 1986, when official national reporting began, over 11 million suspected cases and over 5,000 confirmed deaths were reported. Both infection and fatality rates increased during this period. Statistical time-series forecasting tools, such as the seasonal autoregressive integrated moving average (SARIMA) model, employ a data-centric approach and leverage the highly autocorrelated nature of dengue to predict future incidence.

Machine learning (ML) models have also been applied to forecast dengue in different contexts, often incorporating climate data. In a study by Andersson et al., dengue risk was assessed even more granularly in Rio de Janeiro, Brazil. The authors used convolutional neural networks based on aerial and street-view images to predict dengue risk at the neighborhood level. The potential of machine learning models to predict dengue 1 month ahead in over 200 Brazilian cities was assessed. Different algorithms, including decision tree ensemble approaches, neural networks, SVR, and a seasonal naive baseline model, were compared. The study demonstrated that different models worked best in different cities, and a random forest model trained on monthly dengue cases performed best overall. They were able to predict monthly dengue cases in Brazilian cities 1 month ahead, using data from 2007 to 2019 (45).

This case represents the AI Applications domain and exemplifies Principle 4.2.1 (Context Sensitivity). The SARIMA and random forest models were trained on local epidemiological and meteorological variables to ensure regional accuracy, demonstrating how context-specific applications can mitigate the risks of data mismatch in LMIC settings.

### 6.2 India: TB screening algorithms

India has the world's largest TB burden and accounts for over one quarter of the 3.8 million “missing cases” which go undiagnosed each year^.^ This gap is largely due to the lack of accurate, rapid, and cost-effective tools for TB screening and diagnosis. Now, with the advent of digital CXR, there is renewed interest in using CXR interpreted by computer-aided detection (CAD) software programs for PTB detection. In 2018, a new commercial CAD software with the capacity for PTB detection, *qXR* (Qure.ai, Mumbai, India), received CE certification^.^ According to the company, the software is compatible with all radiology information technology (IT) systems, integrates easily with radiology workflow, and can process one CXR in 10 milliseconds. The pretest probability of PTB in the study was 34%. The AUC achieved by *qXR* for detecting microbiologically-confirmed PTB was 0.81 (95% CI: 0.78, 0.84). The threshold that maximized the sensitivity and specificity of *qXR* simultaneously was 0.818. Using a threshold of 0.818, *qXR* achieved a sensitivity of 71% (95% CI: 66%, 76%) and a specificity of 80% (95% CI: 77%, 83%) (54).

The qXR implementation addresses both the Governance and Implementation domains while demonstrating Principle 4.2.3 (Frugal Innovation). By ensuring compatibility with existing radiology IT systems and processing images in 10 milliseconds, it bypasses the infrastructure and specialized personnel shortages identified in our results, showing how frugal innovation can bridge governance gaps in low-resource settings Table 5. Case Examples of AI in LMICs.

**Table 5:** Case examples of AI in LMICs.

Country/Region	AI Application	Outcome/Impact	Reference
Brazil	Machine learning models for dengue outbreak prediction	Improved early detection and resource allocation; random forest models most accurate	Roster et al. (45)
India	AI-based chest x-ray (qXR) for TB detection	AUC 0.81; sensitivity 71%, specificity 80%; reduced diagnostic delays	Nash et al. (54)
Kenya	OpenMRS electronic health records for maternal/child health	Improved completeness of health data and continuity of care	Haskew et al. (51)

To address the Privacy Domain, the regulatory benchmarks of South Africa's POPIA and India's Digital Personal Data Protection Act serve as practical models. These align with Principle 4.2.2 (Participatory Design) by embedding transparency and user-consent requirements into the legal architecture, ensuring that digital health expansion does not compromise patient rights in resource-limited settings (19, 20).

## 7 Future directions and research gaps

The growing use of AI in underdeveloped nations' healthcare systems poses particular implementation, ethical, and legal issues. In addition to critical reflection, problem-solving necessitates proactive strategies that fill in current gaps and define future research directions.

### 7.1 Context-aware AI development

The majority of AI health applications were developed in high-resource environments, which limits their performance and adaptability in LMICs (55). AI systems must adjust to local epidemiological profiles, linguistic diversity, health system capacities, and sociocultural contexts (55). Strong local demographic databases and participatory design procedures that involve frontline staff and community stakeholders are required for this (56). Methods for developing AI systems that are not only technically sound but also socially and environmentally sound must be further investigated in research (56).

### 7.2 Gender and equity-focused algorithm audits

According to Obermeyer et al., current AI systems have demonstrated a propensity to replicate or even magnify preexisting racial, gender, and socioeconomic biases, resulting in disparate health outcomes (57). Systematic algorithmic audits with specific consideration for intersectionality and equity are desperately needed (57). Impact analyses on marginalized populations, gender-disaggregated data, and continuous monitoring frameworks customized for LMIC settings are all examples of this (58). Standard criteria and approaches for detecting and resolving disparities in algorithmic performance across various demographic groupings should be the top priority of research (58).

### 7.3 AI ethics and law co-development

A notable regulatory obstacle in LMICs is the absence of flexible legal frameworks that change in tandem with advancements in AI (59). Laws frequently either establish broad prohibitions that hinder innovation or lag (59). It is essential to co-develop ethical standards and legal documents through multi-stakeholder engagement among ethicists, legal scholars, public health specialists, and technologists (59). Future research should look at ways to include ethical concepts that are pertinent to the setting, such as accountability, justice, and solidarity, into legislative frameworks that are both enabling and protective (59).

### 7.4 Global south–led research and innovation

The Global North's institutions and narratives dominate a large portion of the global conversation on AI and digital health. Epistemic marginalization and digital dependency could be increased by this imbalance (60). Important steps include decolonizing digital health research, funding regional centers of excellence, and encouraging South-South cooperation (60). To create AI-driven health systems that are really inclusive, ethical, and sustainable, local researchers, technologists, and legislators must be given the authority to lead innovation and establish research priorities (60). When reforming research financing, the Global South should prioritize capacity building and long-term collaborations (60).

## 8 Limitations

This review has several limitations that should be acknowledged. First, as a scoping review, the approach was intentionally non-exhaustive and relied on purposive identification of representative and influential literature rather than comprehensive systematic searching. Consequently, some relevant studies or policy documents may not have been captured. Second, the review was restricted to English-language publications, which may have resulted in underrepresentation of locally produced evidence from LMICs published in other languages. Third, the review draws largely on secondary literature and policy analyses, and no formal risk-of-bias or quality appraisal tools were applied; instead, source credibility was assessed conceptually based on peer-review status, institutional authority, and relevance. Finally, quantitative figures reported across included studies and policy sources reflect heterogeneous contexts and methodologies and should be interpreted as indicative trends rather than precise or universally generalizable estimates. Despite these limitations, the scoping review approach provides valuable conceptual and policy-relevant insights into ethical, regulatory, and implementation challenges shaping equitable AI deployment in LMIC health systems.

## 9 Conclusion

To transition from digital dependency toward inclusive health systems, LMICs must operationalize the four Principles of Inclusive AI established in this review. Specifically, prioritizing Context Sensitivity (Principle 4.2.1) ensures AI is calibrated to local data, while fostering Local Innovation Ecosystems (Principle 4.2.4) reduces reliance on external technologies. The adoption of Participatory Design (Principle 4.2.2) and Open-Source Innovation (Principle 4.2.3) provides the necessary pathways to bridge the identified infrastructure gaps.

AI holds great promise for improving healthcare delivery in developing regions, yet its impact will remain constrained unless structural, ethical, and regulatory barriers are addressed. Challenges such as algorithmic bias, weak data protection, and unclear accountability intersect with fragile legal systems and under-resourced health infrastructures, risking deeper inequality rather than progress. Sustainable advancement requires technologies tailored to local contexts, co-created with regional experts, and aligned with public health priorities. Legal and policy frameworks must embed fairness, transparency, and accountability, while investments in local capacity through professional training, technical education, and inclusive governance ensure digital tools serve real community needs.

Meaningful transformation depends on cross-border collaboration, equitable funding, and active public engagement. Thoughtfully designed and responsibly implemented, AI can strengthen health systems and advance universal health coverage; introduced without safeguards, it risks reinforcing exclusion. The goal is not merely to adopt new technologies, but to integrate them in ways that uphold justice, inclusion, and shared responsibility in public health.

## Data Availability

The original contributions presented in the study are included in the article/Supplementary Material, further inquiries can be directed to the corresponding author.
