# Supplementary material for: Navigating ethical, regulatory, and implementation barriers to AI in healthcare: pathways toward inclusive digital health in low-resource settings—a scoping review
Source: Front Digit Health. 2026 Apr 13;8:1763884. doi: 10.3389/fdgth.2026.1763884 (PMC13111294; doi:10.3389/fdgth.2026.1763884)
Supplement: Supplementary file 1 [file table1.docx]

**Supplementary Table 1: Summary of Evidence Sources (N=60)**

| **No.** | **Source / Study** | **Domain** | **Key Finding / Focus** |
| --- | --- | --- | --- |
| 1 | Wang & Preininger (2019) | Applications | State of the art and future of health AI. |
| 2 | Maleki V. & Forouzanfar (2024) | Applications | AI transforming 21st-century clinics. |
| 3 | Aung et al. (2021) | Applications | Challenges of knowledge augmentation. |
| 4 | Guo & Li (2018) | Applications | AI success in rural LMIC areas. |
| 5 | Schwalbe & Wahl (2020) | Governance | Future strategy for global health AI. |
| 6 | Arawi et al. (2024) | Ethics | 4th Industrial Revolution impact in LMICs. |
| 7 | Tricco et al. (2018) | Methods | PRISMA-ScR methodological guidelines. |
| 8 | Celi et al. (2022) | Privacy/Ethics | Bias and healthcare disparities review. |
| 9 | Nazer et al. (2023) | Privacy/Ethics | Recommendations for bias mitigation. |
| 10 | Fletcher et al. (2020) | Privacy/Ethics | Fairness and appropriate use in global health. |
| 11 | Hewarathna (2025) | Privacy | Cross-border data sharing challenges. |
| 12 | Kiseleva et al. (2022) | Governance | Transparency as a multilayered system. |
| 13 | AI & Society (2025) | Governance | Analysis of opacity and clinical liability. |
| 14 | Alami et al. (2020) | Governance | Sustainable and inclusive AI foundations. |
| 15 | Demaidi (2025) | Governance | National AI strategy for developing nations. |
| 16 | Leam (2023) | Governance | 7.4% strategy adoption rate baseline. |
| 17 | WHO (2021/2026) | Governance | Global strategy on digital health. |
| 18 | Noyes (2025) | Governance | AI for humanity in Africa/Latin America. |
| 19 | POPIA (South Africa) | Privacy | Personal information protection regulatory act. |
| 20 | DPDP Act (India) | Privacy | Digital Personal Data Protection Act 2023. |
| 21 | López et al. (2022) | Applications | Transforming ecosystems through health AI. |
| 22 | Int. Growth Centre (2025) | Governance | Making AI work in LMIC governments. |
| 23 | Ferlito et al. (2024) | Ethics | Ubuntu-inspired responsibility gaps. |
| 24 | Smith (2021) | Governance | Clinical AI liability and accountability. |
| 25 | Maliha et al. (2021) | Governance | Safety vs. innovation in medical AI liability. |
| 26 | TechTarget (2025) | Governance | Health system oversight and governance. |
| 27 | Reddy et al. (2020) | Governance | Healthcare AI governance model. |
| 28 | Kim et al. (2023) | Governance | Organizational governance of AI adoption. |
| 29 | Bellemo et al. (2019) | Applications | Diabetic retinopathy screening in Africa. |
| 30 | Malaquias & Albertin (2019) | Implementation | Infrastructure barriers to tech advancement. |
| 31 | He et al. (2019) | Applications | Practical implementation of medical AI. |
| 32 | Albahar et al. (2023) | Implementation | CDSS barriers in Jordanian hospitals. |
| 33 | Li et al. (2024) | Applications | Personalized healthcare AI innovation. |
| 34 | Ahmed et al. (2023) | Implementation | Systematic review of AI barriers. |
| 35 | CHI Conference (2021) | Implementation | "Brilliant AI Doctor" rural deployment risks. |
| 36 | Fan et al. (2021) | Applications | Case study of self-diagnosis chatbots. |
| 37 | Frimpong (2024) | Privacy/Ethics | Cultural influences on AI apprehension. |
| 38 | Desai (2024) | Applications | AI in pharmacovigilance opportunities. |
| 39 | Carrell et al. (2017) | Applications | Adapting clinical NLP to diverse settings. |
| 40 | Wahl et al. (2018) | Applications | AI contribution to resource-poor settings. |
| 41 | Kasneci et al. (2023) | Applications | ChatGPT and LLM opportunities/challenges. |
| 42 | Nwankwo et al. (2024) | Applications | Telemedicine and AI in rural settings. |
| 43 | Behara et al. (2022) | Applications | South African medical diagnostics review. |
| 44 | Giri & Gupta (2024) | Applications | Disease surveillance through AI. |
| 45 | Roster et al. (2022) | Applications | Dengue fever forecasting in Brazil. |
| 46 | Yang et al. (2024) | Applications | Generalizability of AI across income tiers. |
| 47 | Sathitratanacheewin(2018) | Applications | Specificity limits in automated TB reading. |
| 48 | Weissglass (2022) | Privacy/Ethics | Contextual bias and democratization. |
| 49 | Najjar (2023) | Applications | Radiology and AI integration review. |
| 50 | Hosny & Aerts (2019) | Applications | AI for global health risks and benefits. |
| 51 | Haskew et al. (2015) | Governance | Cloud-based EMR for maternal health (Kenya). |
| 52 | Bazzano et al. (2025) | Governance | Community engagement in public health AI. |
| 53 | Sieber et al. (2025) | Governance | Civic participation in AI development. |
| 54 | Nash et al. (2020) | Applications | Radiography reading for TB in India. |
| 55 | Abebe et al. (2020) | Privacy/Ethics | Roles for computing in social change. |
| 56 | Naik et al. (2022) | Governance | Legal/ethical responsibility in AI surgery. |
| 57 | Obermeyer et al. (2019) | Privacy/Ethics | Dissecting racial bias in health algorithms. |
| 58 | WHO Guidance (2021) | Governance | Ethics and Governance of AI for Health. |
| 59 | Floridi et al. (2018) | Privacy/Ethics | AI4People: Ethical framework for society. |
| 60 | Birhane (2021) | Privacy/Ethics | Relational ethics and algorithmic injustice. |
